# The Warm Bath in Insomnia

**Published:** 1895-08

**Authors:** 


THE WARM BATH IN INSOMNIA.—According to Dr. Eccles, the use of the warm bath for the purpose of producing sleep is very efficient if properly carried out. The bath should be administered in a room whose temperature is 65° to 70° F. The patient is made to stand with his head over the edge of the tub, and his head and face are then rapidly douched with water at 100° F. The cooling of the body by the air and the hot sponging of the head send the blood to the head, dilating the vessels of the entire brain. The entire body is then immersed, except, of course, the head, in a bath at 98° F., which is rapidly raised to a temperature of 105° to 110° F. In a few minutes the patient is taken from the bath, wrapped in warm blankets, and without exertion on his part, taken to his room. The blankets absorb the moisture; in his room the night clothes are put on, a warm bottle placed at his feet, and possibly some warm liquid food administered. The sedative and refreshing result is often most marked.—*The Sanitarian.*

